# Effect of home-based transcranial direct current stimulation combined with nutritional counseling therapy on binge eating disorder symptoms: a randomized pilot trial

**DOI:** 10.47626/1516-4446-2024-3776

**Published:** 2025-07-02

**Authors:** Jessica Lorenzzi Elkfury, Luciana C. Antunes, Betina Franceschini Tocchetto, Lizia Nardi Menegassi, Paulo Sanches, Danton Pereira, Liciane Fernandes Medeiros, Tiago M. Cardinal, Iraci L.S. Torres, Felipe Fregni, Wolnei Caumo

**Affiliations:** 1 Programa de Pós-Graduação em Ciências Médicas, Faculdade de Medicina, Universidade Federal do Rio Grande do Sul (UFRGS), Porto Alegre, RS, Brazil; 2 Laboratório de Dor e Neuromodulação, UFRGS, Porto Alegre, RS, Brazil; 3 Departamento de Nutrição, Centro de Ciências da Saúde, Universidade Federal de Santa Catarina, Florianópolis, SC, Brazil; 4 Laboratório de Engenharia Biomédica, Hospital de Clínicas de Porto Alegre (HCPA), Porto Alegre, RS, Brazil; 5 Programa de Pós-Graduação em Saúde e Desenvolvimento Humano, Universidade La Salle, Canoas, RS, Brazil; 6 Programa de Pós-Graduação em Ciências Biológicas: Farmacologia e Terapêutica, UFRGS, Porto Alegre, RS, Brazil; 7 Núcleo de Pesquisa em Farmacologia da Dor e Neuromodulação, HCPA, RS, Brazil; 8 Spaulding Neuromodulation Center, Department of Physical Medicine and Rehabilitation, Spaulding Rehabilitation Hospital, Charleston, SC, USA; 9 Departamento de Cirurgia, Faculdade de Medicina, UFRGS, Porto Alegre, RS, Brazil

**Keywords:** Binge eating disorder, transcranial direct current stimulation, cortical excitability, nutritional therapy

## Abstract

**Objective:**

To examine the effect of nutritional counseling therapy (NCT) combined with transcranial direct current stimulation (tDCS) on binge eating disorder (BED) symptoms.

**Methods:**

Forty women with BED were randomly allocated (2:2:2 ratio) to one of the following groups: active tDCS (a-tDCS), NCT, sham tDCS (s-tDCS) with NCT, and a-tDCS with NCT. Home-based tDCS was applied to the dorsolateral prefrontal cortex for 28 sessions.

**Results:**

A mixed analysis of variance (ANOVA) showed no main effect between groups nor a time × group interaction. However, a significant main effect was found for time on the primary outcome Binge Eating Scale (BES) (p = 0.001; eta^2^p = 0.325), which tended to decrease during treatment and follow-up. A significant main effect was found for the secondary outcome short-interval intracortical inhibition (SICI) (p = 0.02; eta^2^p = 0.112), a measure of inhibitory function, which increased from baseline to the final period in the a-tDCS group, without significant differences between groups.

**Conclusion:**

Combined NCT and tDCS did not have a synergistic effect on BED symptoms. Nevertheless, the data from this pilot study should help plan future larger-scale studies investigating the effects of tDCS and behavioral interventions in the promising area of BED treatment.

**Clinical trial registration:**

ClinicalTrials.gov: NCT 04226794. Registered on July 2, 2019.

## Introduction

Binge eating disorder (BED) is a psychiatric disorder associated with several medical and mental health problems.^1^,^2^ Despite improvements in understanding the BED diagnosis, its physiopathology is still poorly understood.^2^ The strongest correlation found has been between the severity of BED symptoms and abnormalities in the reward system, particularly those affecting the mesolimbic and mesocortical dopaminergic pathways.^3^ In addition, BED neurobiology includes alterations in executive function (inhibitory control), attention, impulsivity/compulsivity, and decision-making.^3^,^4^

BED management remains challenging, with few effective treatments available.^5^ Despite cognitive behavioral therapy (CBT) being the first-line therapy of choice, major issues remain to be overcome, such as substantial unresponsiveness (25 to 40%) and high dropout rates (7 to 73%). There is a clear need for innovative strategies that can be added to conventional interventions to improve disease-related outcomes. In this regard, neuromodulatory techniques, such as transcranial direct current stimulation (tDCS), might be a useful therapeutic tool, since they may be able to change dysfunctional brain activity patterns and rearrange faulty circuits^6^,^7^ though long-lasting neuroplastic effects.^8^

A meta-analysis on individuals with food cravings, obesity, and drug addiction showed that neuromodulation applied to the bilateral dorsolateral prefrontal cortex (DLPFC) decreased desire and consumption in all samples in both the short and long term (12 months).^9^ Also, previous findings have revealed the feasibility and clinical effects of active tDCS (a-tDCS) on inhibitory control training by improving BED severity when compared to a sham tDCS (s-tDCS) group.^10^ Therefore, preliminary evidence indicates a significant potential of neurostimulation to modulate dysfunctional eating behaviors and body weight. Conversely, several methodological and clinical gaps remain to be explored in randomized clinical trials assessing synergistic effects of tDCS with conventional interventions in BED samples, due to the heterogeneity found in outcome measures, the scarcity of neurophysiological and neuroimaging measurements, the relatively short period of interventions, and the lack of follow-up.^11^,^12^

The use of tDCS as a complementary therapy in BED may be improved by understanding its impact on BED symptoms and neurophysiological surrogate outcomes while accounting for neuroplasticity processes, aiming to associate them with clinical effectiveness. Within this context, we evaluated whether combining 28 home-based sessions of self-applied a-tDCS to the DLPFC with nutritional counseling therapy (NCT) would synergistically reduce BED symptom severity (primary outcome) compared to s-tDCS with NCT, NCT alone, or a-tDCS alone. We also evaluated secondary outcomes associated with inhibitory function, including short-interval intracortical inhibition (SICI) and a Go/No-Go task, and clinical outcomes, including body weight and cognitive and behavioral components of eating and food craving.

## Methods

### Design overview, setting, and participants

#### Study design

**Figure 1:**
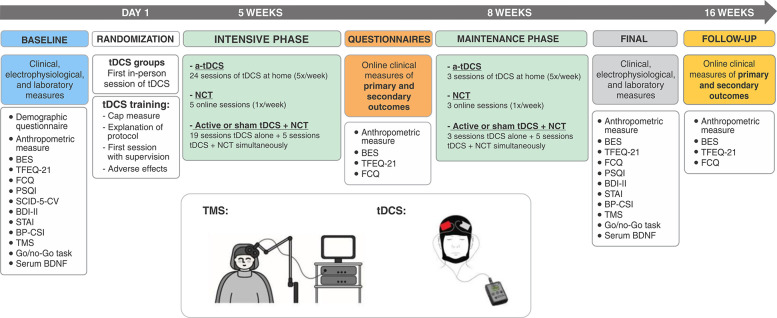
Study protocol. a-tDCS = active transcranial direct current stimulation; BDI-II = Beck Depression Inventory; BDNF = brain-derived neurotrophic factor; BES = Binge Eating Scale; BP-CSI = Brazilian Portuguese-Central Sensitization Inventory; FCQ = Food Craving Questionnaire; NCT = nutritional counseling therapy; PSQI = Pittsburgh Sleep Quality Index; STAI = State-Trait Anxiety Inventory; tDCS = transcranial direct current stimulation; TFEQ-21 = Three Factor Eating Questionnaire; TMS = transcranial magnetic stimulation.

This double-blind, factorial, parallel-group, randomized pilot, sham-controlled trial was conducted according to the principles of the Declaration of Helsinki, the International Council for Harmonization of Good Clinical Practice guidelines, and all applicable Brazilian laws and research regulations. Its protocol was reviewed and approved by the institutional review board of the Hospital de Clínicas de Porto Alegre (HCPA; approval number: 0000921) and registered at ClinicalTrials.gov (ID: NCT 04226794; registration date: July 2, 2019). All participants provided written informed consent before starting trial-related procedures. The data were collected from 2019 to 2021, during the COVID-19 pandemic, and the detailed protocol has been previously reported.^13^ The timeline of the study shown in Figure 1.

#### Participants

The participants were recruited at psychiatric clinics and in the community in Porto Alegre, a large city in Southern Brazil. Eligible participants were adults aged 18-65 years who met the DSM-5 criteria for BED diagnosed using the SCID-5-Clinician Version (SCID-5-CV), conducted by trained psychiatrists. The inclusion criteria were female sex, moderate or severe BED (defined by more than four episodes of binge eating per week), overweight or obesity, literacy, and right-handedness. We have chosen to include only women based on three criteria: i) the prevalence of BED is higher in women; ii) tDCS might have sex-specific impacts on the outcomes of BED; and iii) distinct hormonal profiles between sexes could bias results. The exclusion criteria were pregnancy, night-shift workers (00:00-05:00), treatment for weight loss within the last 30 days, bariatric surgery, inability to perform home-based tDCS, and formal contraindications for tDCS (previous history of neurosurgery, presence of any ferromagnetic metal in the head, implanted medical devices in the head or neck region, and history of uncontrolled epilepsy with seizures in the preceding year).

### Interventions

#### Home-based transcranial direct current stimulation

We used a home-based tDCS device developed and validated by our research group and the HCPA (registration number: NCT02408237) according to leading standards, with safety features for adjusting tolerability and resistance intensity. The minimum interval between consecutive tDCS sessions was 16 hours to avoid excessive use.^14^ Adherence was evaluated through software showing the data of all performed sessions and the current intensity values as the contact impedance of each session.

After the baseline assessment, the participants were allocated to the tDCS groups, instructed about the protocol, and trained to self-apply the home-based tDCS. To assess potential adverse effects, the participants were instructed to correctly complete the adverse effects diary immediately after each tDCS home session. They were also asked about any new adverse events at each virtual appointment.

The device was programmed to deliver a 2-mA active current to the right (anode) and left (cathode) DLPFC, positioned over F4 and F3, respectively, according to the international 10-20 system for electroencephalography. The electrodes were attached to a cap sized appropriately for the participant’s scalp (small, medium, or large), ensuring their position would not change and, thus, guaranteeing accurate placement to deliver the electric current during stimulation. The device came with two silicone cannulas attached to 35-cm^2^ (5 × 7 cm) electrodes coated in sponges soaked with saline solution, through which the current was delivered.

The right DLPFC was the target of the anodal stimulation because it had shown the greatest therapeutic efficacy in previous studies at the time of study conception.^15^ However, it is now known that dysfunctional eating behaviors might result from dysregulation between bilateral DLPFCs, with positive effects from anodal stimulation on both sides.^15^,^16^ The a-tDCS group received the current for 20 minutes. The s-tDCS group received only 30 seconds of stimulation at the beginning, midpoint, and end of the session.

Our skilled research staff assisted participants in the first stimulation session. The subsequent sessions occurred at home on weekdays. Participants were instructed to stay seated and awake during the stimulation period. Additionally, they had scheduled weekly online appointments to check the device’s functioning, and a research contact mobile number was made available 24 hours a day, 7 days a week should they require assistance.

The protocol was subdivided into an intensive phase of 20 sessions (5 days/week) and a maintenance phase of three additional sessions (1 day/week), for a total of 28 sessions. Minimal changes were made to the original protocol due to the restrictions imposed by the coronavirus disease 2019 (COVID-19) pandemic. Therefore, all sessions (except the first) were performed at home and not once weekly at the hospital, as previously described.^13^

#### Nutritional counseling therapy

NCT sessions occurred weekly, for a total of eight virtual sessions, each lasting 40 minutes (20 minutes of recorded video and 20 minutes for activities and feedback about the previous session). This protocol was based on standard CBT^17^ and approached the following topics: psychoeducation on BED; expectations/motivations regarding treatment; identification of feelings and emotions and how they interfere with dieting and eating behavior; feeding neurophysiology, beliefs, trigger situations, and mindful eating; planning strategies to organize food routine and coping cards; and relapse prevention and closure.

### Study protocol and randomization

This study was divided into five phases (Figure 1): 1) baseline data collection before randomization; 2) intensive phase: 20 sessions of tDCS (Monday to Friday) and/or weekly NCT (five appointments); 3) maintenance phase: three sessions of tDCS (weekly) and/or weekly NCT (three appointments); 4) end of study; and 5) follow-up (Figure 1).

After completing the baseline phase, participants were randomly allocated in blocks of eight, using a computer program (2:2:2 ratio), to one of four groups: 1) a-tDCS; 2) NCT only; 3) s-tDCS with NCT; and 4) a-tDCS with NCT. The same evaluator examined all treatment sessions to reduce variability, except for the questionnaires that were virtual forms. The intermediate data were collected exclusively using virtual forms. The same evaluator performed all treatment appointments, which were virtual except for the baseline and end-of-study appointments.

### Outcomes

Participants were assessed at baseline, at 5 and 8 weeks during the treatment phase, and at 16 weeks during the follow-up phase. Their sociodemographic information and medical history, including clinical and psychiatric comorbidities, were collected using a standardized questionnaire.

The primary outcomes were changes in the severity of binge eating symptoms as measured with the Binge Eating Scale (BES), a self-administered questionnaire widely used as a dimensional measure of the severity of binge eating, a screening tool, and a helpful instrument to assess treatment outcomes.^18^-^20^ Its score ranges from 0 to 46 points, where ≤ 17 points indicates minimal binge eating behavior, 18-26 points indicates moderate binge eating behavior, and ≥ 27 points indicates severe binge eating behavior.^19^ The BES version adapted and validated for the Brazilian population is considered suitable for clinical use.^19^

The secondary outcomes were the SICI, a surrogate measure of cortical excitability measured by paired-pulse transcranial magnetic stimulation (TMS); inhibitory control of executive function, assessed using a Go/No-Go task; body weight; and eating behavior psychopathology, measured using the Three Factor Eating Questionnaire-21 (TFEQ-21)^21^ and the Food Craving Questionnaire (FCQ).^22^ The same evaluator assessed all measures.

Eating behavior, especially BED, is influenced by several variables that can act as confounding factors in studies, such as sleep quality, which was evaluated using the Pittsburgh Sleep Quality Index (PSQI),^23^ and mental health, which was evaluated using the SCID-5-CV in an interview with a trained psychiatrist.^24^ Similarly, depressive symptoms were evaluated through the Beck Depression Inventory-II (BDI-II),^25^ and anxiety was evaluated using the State-Trait Anxiety Inventory (STAI).^26^ We also administered the Brazilian Portuguese-Central Sensitization Questionnaire (BP-CSI), since central sensitization has been associated with the pathophysiology of psychosomatic conditions such as eating disorders.^27^,^28^ Brain-derived neurotrophic factor (BDNF) levels were also measured due to their important role in regulating neuronal survival, growth, and differentiation^29^ and their ability to predict the therapeutic response to tDCS.^30^

### Sample size

Since no earlier studies had evaluated the combination of tDCS and NCT, we conducted a pilot assessment with a sample size chosen according to the recommendations of Kieser and Wassmer,^31^ i.e., between 20 and 40 subjects for standardized effect sizes of 0.4-0.7 and a type II error rate of 90%. Based on this assumption, we included 40 patients.^32^,^33^

### Blinding

Two independent researchers who were not involved in evaluating the participants performed the randomization before data collection began. The random number sequence was delivered in sealed envelopes by the staff of the Biomedical Engineering Division, who were responsible for setting up the tDCS devices.

Throughout the protocol, no one knew the tDCS programming (active or sham), including study employees, investigators engaged in patient care, and those who administered the scales. Two biomedical engineers (PRS and DPS) who were not involved in evaluating the participants prepared the tDCS devices to provide either active or sham stimulation according to the randomization code. The validity of sham stimulation was assessed with the participants through a blinding questionnaire in the follow-up phase. Unblinding was performed once all data collection had ended.

### Statistical analysis

Values are presented descriptively as the mean (SD), median (interquartile range), or frequency (percentage). The normality of the continuous variables was assessed using the Shapiro-Wilk test. Baseline characteristics and delta values of the various groups were evaluated by descriptive analyses and compared using analysis of variance (ANOVA) for parametric variables or the Kruskal-Wallis rank order test for non-parametric variables. Correlations between variables were assessed using Pearson’s product-moment correlation coefficient (*r*). In the pre-specified analysis plan, the endpoint related to binge eating symptoms (measured by the BES) and the secondary outcomes (SICI, weight, TFEQ-21, FCQ, and Go/No-Go task) were analyzed using a mixed effects model for repeated measures, considering phase and treatment as fixed effect factors, and subjects within the sequence and within-subject error as random factors.

All participants were included in an intention-to-treat (ITT) analysis, with the expectation-maximization algorithm used to manage missing data, accounting for the pattern of participants’ responses to create a better estimator. The effect size between groups was estimated using Cohen’s *d* and defined cutoffs for small, medium, and large effect sizes of 0.2, 0.5, and 0.8, respectively.^34^ Two-sided 95%CI were calculated for all observed between-group differences, and a p < 0.05 was considered statistically significant. All analyses were performed in SPSS version 28.0.

## Results

### Participants’ demographic and clinical characteristics

**Figure 2:**
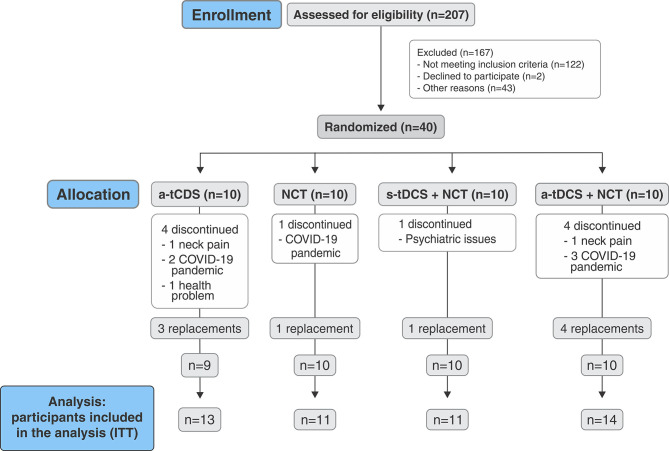
Study flowchart. a-tDCS = active transcranial direct current stimulation; COVID = coronavirus disease; ITT = intention-to-treat; NCT = nutritional counseling therapy; s-tDCS = sham transcranial direct current stimulation.

Of the 207 screened patients, 167 did not meet the criteria for inclusion. The other 40 were enrolled in the study and randomized equally into the four groups (n=10 per group): 1) a-tDCS only; 2) NCT only; 3) s-tDCS + NCT; and 4) a-tDCS + NCT. Thirty participants (75%) completed the trial: six (60%) in the a-tDCS group, nine (90%) in the NCT group, nine (90%) in the s-tDCS + NCT group, and four (60%) in the a-tDCS + NCT group. Of the 10 participants who dropped out, six discontinued therapy due to the COVID-19 pandemic, two due to neck pain associated with the treatment (a-tDCS and a-tDCS + NCT groups), one due to psychiatric issues (s-tDCS + NCT group), and one due to other health problems (a-tDCS group). To address this appreciable loss, we replaced nine participants after the planned collections had ended, while respecting the blinded randomization (Figure 2).

Table 1 presents the participants’ epidemiological and clinical characteristics at baseline by treatment group. As mentioned above, we conducted an ITT analysis, so all participants were included according to their randomized treatment assignment.

### Primary outcome: changes in binge eating severity assessed using the Binge Eating Scale

**Figure 3:**
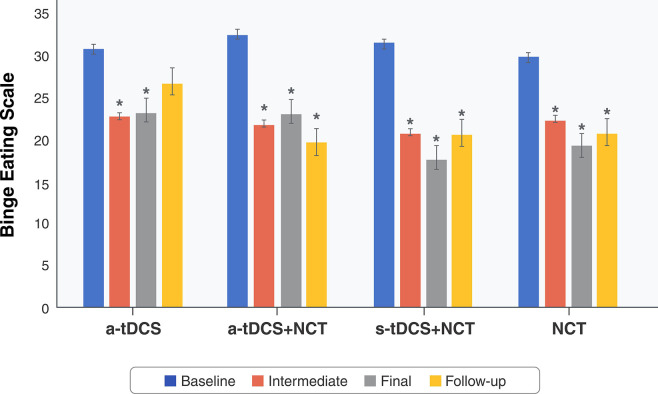
Changes in BES scores over time by treatment group (n=49). Mixed ANOVA was used to compare BES scores between groups and among data collection time points within groups (baseline, intermediate, final, and follow-up). A significant main effect was found for BES (p < 0.001), with BES scores tending to decrease with time, but not between interventions. ANOVA = analysis of variance; a-tDCS = active transcranial direct current stimulation; BES = Binge Eating Scale; NCT = nutritional counseling therapy; s-tDCS = sham transcranial direct current stimulation. * Significant within-group difference from baseline (p < 0.05).

No variables significantly predicted the BES score (p = 0.12). A mixed ANOVA revealed a significant main effect for time on BES (p < 0.001; eta^2^p = 0.325; power = 1.0), with BES scores tending to decrease over time. The intervention showed no main effect (p = 0.59), and no interaction achieved significance (p < 0.36). Post-hoc Bonferroni-corrected tests showed significant differences among the intervention groups during the study period. Compared to the baseline measurements, BES scores improved in the four groups over time (p < 0.05 for all-time points). All but the a-tDCS group (p = 1.00) maintained these results at follow-up (p < 0.05 for all time points). No differences were found across other combinations (Figure 3). Table 2 shows the mean BES scores in each group and ANOVA assessments of the differences in delta values between the baseline and final phases.

### Secondary outcomes

Table 3 presents the mean values for the secondary outcomes in each group and ANOVA assessments of differences in delta values between the baseline and final phases.

#### Anthropometric measure

The mixed ANOVA indicated no significant main effect for time on weight (p = 0.15), no main effect for the intervention on weight (p = 0.12), and no group × time interaction (p = 0.05).

#### Assessment of the severity of eating psychopathology symptoms

**Figure 4:**
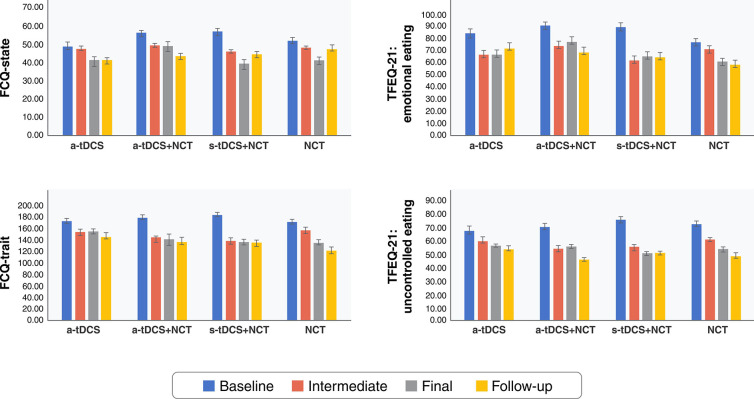
Differences in eating psychopathology over time treatment by group (n=49).

Food craving was evaluated through the FCQ-trait and FCQ-state questionnaires. The mixed ANOVA revealed only a statistical difference in the main effects of time for both questionnaires (p < 0.001) (Figure 4). The post-hoc Bonferroni-corrected tests revealed that FCQ-state scores decreased from baseline to follow-up in the a-tDCS + NCT group (p ≤ 0.01). The FCQ-state scores also decreased in the s-tDCS + NCT group across the four time points (p < 0.05 for all-time points). In contrast, only baseline and final FCQ-state scores differed significantly in the NCT group (p ≤ 0.01). No differences were found across other combinations.

Mixed ANOVA compared FCQ-state, FCQ-trait, and TFEQ-21 emotional and uncontrolled eating scores between groups and across data collection time points within groups (baseline, intermediate, final, and follow-up). A significant main effect was found for the four subscales (p < 0.001), with their scores tending to decrease over time, but not between interventions. ANOVA = analysis of variance; a-tDCS = active transcranial direct current stimulation; FCQ = Food Craving Questionnaire; NCT = nutritional counseling therapy; s-tDCS = sham transcranial direct current stimulation; TFEQ-21 = Three Factor Eating Questionnaire.

The a-tDCS + NCT and s-tDCS + NCT groups showed similar differences in FCQ-trait scores, with symptoms improving from baseline to the intermediate, final, and follow-up phases (p < 0.05 for all time points). In addition, the FCQ-trait scores differed significantly between the baseline and the final and follow-up phases and between the intermediate and follow-up phases in the NCT group (p < 0.05 for all time points).

Eating behavior was examined using the TFE-uncontrolled and TFE-emotional eating subscales (Figure 4). The mixed ANOVA revealed a statistical difference in the main effects for both subscales (p < 0.001). The post-hoc Bonferroni-corrected tests showed that scores for the emotional eating-related domain (TFEQ-21) decreased from baseline to the intermediate and final phases in the a-tDCS group (p < 0.05 for all time points). Similarly, they decreased from baseline to the intermediate and follow-up phases in the a-tDCS + NCT and s-tDCS + NCT groups (p < 0.05 for all time points). In the NCT alone group, they only differed significantly between the baseline and follow-up phases (p = 0.03) and between the intermediate to follow-up phases (p < 0.05 for all time points).

Scores in the uncontrolled eating-related domain (TFEQ-21) likewise decreased from baseline to the intermediate and follow-up phases in the a-tDCS + NCT group and between baseline and all four subsequent phases in the s-tDCS + NCT group (p < 0.05 for all time points). In contrast, they only differed significantly between the baseline and follow-up phases in the NCT group (p ≤ 0.01).

#### Changes in electrophysiological measures of inhibitory control

A mixed ANOVA examined the measures of cortical excitability, such as SICI evaluated through the TMS and cognitive tasks and the number of commission errors in the Go/No-Go task (the so-called accuracy of false alarms, ACCFA), representing the number of incorrect answers during the No-Go trials for food and neutral stimulus.

The mixed ANOVA indicated a main effect for the SICI (p = 0.02; eta^2^p = 0.112; power = 0.6), with post-hoc Bonferroni-corrected tests indicating a significant increase in SICI from baseline to the final phase in the a-tDCS group. However, it indicated no intervention effect (p = 0.62) or time × group interaction (p = 0.16).

In the Go/No-Go task, the mixed ANOVA results were similar for the food and neutral stimuli. It indicated no main effect for time, intervention, or the time × group interaction with the food stimulus (p = 0.43, 0.75, and 0.43, respectively) or the neutral stimulus (p = 0.21, 0.29, and 0.75, respectively).

#### Assessment of adherence and adverse events

We measured adherence by counting the number of completed sessions, as verified by the software records. The median (interquartile range) number of sessions administered was 23.5 (12.7-27.5) in the a-tDCS group, 24.0 (2.5-28.0) in the a-tDCS + NCT group, and 27.5 (25.7-28.0) in the s-tDCS + NCT group. The total number of sessions was 364 in the a-tDCS group (n=13), of which 252 were valid (69.2%); 392 in the a-tDCS + NCT group (n=14), of which 267 were valid (68.1%); and 308 in the s-tDCS +NCT group (n=11), of which 275 were valid (89.3%). When considering only those participants who concluded treatment, the adherence was 89.3% in the a-tDCS group (n=9), 92.9% in the a-tDCS + NCT group (n=10), and 97.9% in the s-tDCS + NCT group (n=10).

## Discussion

This randomized pilot study evaluated whether combining NCT with 28 home-based sessions of self-applied a-tDCS to the DLPFC would synergistically reduce BED symptom severity compared to s-tDCS with NCT, NCT alone, or a-tDCS alone. We have shown that both NCT and a-tDCS were able to reduce BED symptoms, without difference from the combined treatment. Besides the reductions in BED symptoms observed in all groups, changes in cortical excitability were only observed in the a-tDCS group. Altogether, these findings contradict our initial hypotheses, which predicted that combining NCT with tDCS would enhance its efficacy in treating BED.

These results are relevant to understanding how neuroplasticity might be changed by such therapeutic approaches in BED. Nonetheless, our findings must be interpreted cautiously because they were obtained from a pilot study, and type II errors cannot be excluded. It is also possible that the absence of a synergistic effect results from metaplasticity (i.e., when the effect of one plasticity protocol modulates the other plasticity protocol if the two are used together).^35^ Depotentiation, which refers to two protocols that do not induce excitability changes alone but cancel each other out when used together to achieve homeostasis, is another mechanism that could explain this finding.^31^,^36^,^37^ An inverted U-shaped dose-effect curve, where a decrease follows an initial increase, is another possible explanation for this finding.^38^

Few studies have evaluated concurrent use of tDCS, cognitive training, and CBT. Their results likewise did not show additional improvement from combining tDCS with CBT.^39^,^40^ However, this is a promising area of research that could be clinically applicable to developing a treatment plan for BED and confirming or refuting whether combining NCT with a-tDCS improves effectiveness in treating binge eating.^41^ Therefore, it makes sense to suggest larger trials to address concerns about our negative results, which were possibly due to our modest sample size. Consistent with this perspective is the theoretical assumption that tDCS might improve the circuit involved in BED^42^ in the default mode networks related to executive control.^43^

This trial had key methodological differences from previous studies of tDCS in BED. First, it used a home-based tDCS device that enabled considerably more sessions to take place, with 28 sessions being the highest number applied in BED studies to date. This difference is particularly relevant since preliminary evidence indicates that tDCS efficacy increases with treatment length.^44^-^47^ Our results support this view since they show a positive treatment effect on BED after 28 tDCS sessions, despite the lack of difference among group interventions. Second, when interpreting our results, we must consider the positioning of the electrodes, since we applied the anode to the right DLPFC and the cathode to the left DLPFC. While there is no agreement on the best positions for BED, studies have reported variable effects, showing significant improvements in bilateral DLPFC despite laterality.^16^,^47^

Additionally, as demonstrated in the within-group analysis, a-tDCS increased the SICI, a measurement related to cortical inhibition (Table 3). This surrogate outcome highlights the neurophysiological effect of a-tDCS. Despite this discrepancy between the SICI measure related to the inhibitory system and clinical symptoms associated with binge eating in our study, the tDCS effect has been observed in individuals with addiction.^43^,^48^,^49^ While studies using tDCS in the addiction field have produced some preliminary evidence on combining a-tDCS over the DLPFC with cognitive bias modification training, this issue must be further studied to establish a definitive conclusion.^50^,^51^ Similarly, there is evidence of the positive effect of combining tDCS with a concurrent psychotherapeutic intervention in patients with post-traumatic stress disorder and mood disorders.^52^ Despite a plausible biological basis for the effects of tDCS and NCT on the mesocorticolimbic system and inhibitory control, the literature on tDCS effects on binge eating is mixed and remains inconclusive. Further studies with larger sample sizes are needed to generate consistent evidence.

Our study had several limitations. First, it was a pilot study that is sensitive to potential type II errors, which could mask significant treatment differences. Despite its modest sample size, it contributes to the knowledge in the field and to future clinical trial designs. Second, the study occurred during the COVID-19 pandemic, which significantly impacted data collection and dropout rates. While we cannot exclude the importance of this limitation in the study design, we replaced participants who dropped out while respecting the randomization. Also, a lack of an isolated sham tDCS group might be an important limitation to address, due the undeniable effects expected by the subjects, which we attempted to minimize through appropriate blinding.

However, we believe that our findings are reliable for understanding differences in the neurophysiological process underlying the effects of a-tDCS compared to NCT. These findings extend the literature on the distinct neurobiological processes underpinning a-tDCS and NCT; however, they cannot support therapeutic decision-making in a clinical setting.

Our findings show that the assessed combined therapy protocol did not synergically affect BED symptoms and that the effects of NCT might reduce BED symptoms even after treatment ends. In contrast, tDCS increased inhibitory function in the cortex and temporarily reduced BED symptoms. Overall, these findings provide data to inform future, larger-scale studies to investigate the effects of tDCS and NCT.

## Disclosure

The authors report no conflicts of interest.

## Figures and Tables

**Table 1:** Participants’ epidemiological and clinical characteristics at baseline by treatment group (n*=*49)

	a-tDCS	a-tDCS + NCT	s-tDCS + NCT	NCT	
Characteristic	(n=13)	(n=14)	(n=11)	(n=11)	p-value
Demographic					
Age (years)†	35.2 (6.2)	32.0 (7.8)	33.7 (9.6)	31.0 (8.8)	0.61
BMI (kg/m^2^)‡	31.4 (6.4)	35.1 (4.7)	32.5 (3.9)	32.1 (4.0)	0.11
Education level (years)†	17.5 (2.1)	15.5 (3.0)	16.0 (4.7)	15.3 (2.4)	0.34
Clinical					
BDI-II†	26.0 (10.4)	29.5 (10.9)	22.5 (14.7)	20.0 (9.5)	0.20
STAI-State‡	34.6 (7.1)	34.9 (6.9)	42.1 (20.3)	34.5 (5.6)	0.40
STAI-Trait‡	25.3 (2.7)	25.9 (4.7)	23.7 (8.9)	24.8 (7.4)	0.93
BP-CSI†	46.8 (21.4)	43.0 (20.1)	41.3 (25.1)	38.6 (17.8)	0.83
PSQI‡	7.3 (4.4)	8.8 (3.2)	8.7 (4.6)	6.4 (3.4)	0.26
Biochemical					
Serum BDNF†	34.5 (14.0)	43.7 (12.1)	42.1 (12.0)	38.7 (11.2)	0.26

Data presented as mean (SD).

a-tDCS = active transcranial direct current stimulation; BDI = Beck Depression Inventory-II; BDNF = brain-derived neurotrophic factor; BMI = body mass index; BP-CSI = Brazilian Portuguese-Central Sensitization Inventory; NCT = nutritional counseling therapy; PSQI = Pittsburgh Sleep Quality Index; STAI = State-Trait Anxiety Inventory; s-tDCS = sham transcranial direct current stimulation.

† Parametric variables were compared using analysis of variance (ANOVA).

‡ Non-parametric variables were compared using the Kruskal-Wallis rank order test.

**Table 2:** Mean differences in BES scores from before to after the intervention (n=49)

Primary outcome/Treatment	Mean (SD) before vs. after treatment	Mean difference (95%CI)†	p-value‡
BES			
a-tDCS	30.67 (7.49) vs. 23.45 (7.13)	7.21 (1.50-12.92)	0.033
a-tDCS + NCT	32.64 (6.00) vs. 23.37 (11.45)	9.27 (3.90-14.63)	0.001
s-tDCS + NCT	31.42 (8.15) vs. 17.82 (8.35)	13.59 (8.49-18.69)	0.001
NCT	30.09 (4.23) vs. 19.37 (7.67)	10.72 (5.63-15.81)	0.001

a-tDCS = active transcranial direct current stimulation; BES = Binge Eating Scale; NCT = nutritional counseling therapy; s-tDCS = sham transcranial direct current stimulation.

† Mean difference from before to after the intervention.

‡ Bonferroni-corrected p-value for the within-group pre- to post-intervention effect from a mixed analysis of variance (ANOVA).

**Table 3:** Mean differences in secondary outcomes from before to after the intervention (n=49)

Secondary outcome/Treatment	Mean (SD) before vs. after treatment	Mean difference (95% CI)	p-value†
Weight‡			
a-tDCS	82.24 (18.02) vs. 85.45 (18.29)	1.79 (-2.16 to 5.73)	1.000
a-tDCS + NCT	94.03 (13.38) vs. 103.73 (16.61)	-9.70 (-19.40 to 0.02)	0.005
s-tDCS + NCT	88.81 (12.44) vs. 87.52 (11.62)	1.28 (-0.32 to 2.90)	1.000
NCT	87.02 (11.79) vs. 91.10 (13.06)	-4.08 (-11.24 to 3.08)	0.658
FCQ-trait§			
a-tDCS	171.33 (30.98) vs. 153.12 (30.30)	18.21 (3.49 to 32.94)	0.281
a-tDCS + NCT	176.07 (19.52) vs. 140.09 (32.45)	35.98 (15.37 to 56.59)	<0.001
s-tDCS + NCT	182.33 (22.94) vs. 135.49 (31.54)	46.84 (26.35 to 67.34)	<0.001
NCT	170.27 (23.83) vs. 133.80 (40.37)	36.48 (16.36 to 56.59)	0.002
FCQ-state§			
a-tDCS	48.08 (7.30) vs. 40.63 (10.52)	7.45 (3.14 to 11.77)	0.082
a-tDCS + NCT	55.18 (7.51) vs. 48.56 (13.13)	6.62 (0.23 to 13.01)	0.106
s-tDCS + NCT	55.92 (6.63) vs. 39.10 (12.04)	16.81 (9.60 to 24.02)	<0.001
NCT	51.27 (9.35) vs. 40.79 (13.66)	10.48 (3.67 to 17.30)	0.007
TFEQ-21: uncontrolled eating§			
a-tDCS	67.00 (12.48) vs. 56.48 (16.49)	10.52 (0.44 to 20.60)	0.632
a-tDCS + NCT	69.55 (16.43) vs. 56.02 (25.98)	13.53 (-3.22 to 30.29)	0.158
s-tDCS + NCT	75.02 (15.80) vs. 50.82 (19.05)	24.19 (11.10 to 37.29)	0.003
NCT	72.08 (12.86) vs. 53.83 (17.83)	18.25 (5.70 to 30.79)	0.052
TFEQ-21: emotional eating§			
a-tDCS	83.81 (20.29) vs. 68.04 (22.20)	15.77 (0.55 to 30.98)	0.035
a-tDCS + NCT	91.09 (13.61) vs. 78.50 (25.00)	12.59 (2.19 to 22.99)	0.098
s-tDCS + NCT	90.74 (12.61) vs. 66.78 (20.29)	23.96 (13.63 to 34.29)	<0.001
NCT	77.26 (22.41) vs. 62.88 (26.30)	14.38 (3.48 to 25.28)	0.091
Go/No-Go: ACCFA-Neutral§			
a-tDCS	0.13 (0.09) vs. 0.12 (0.08)	< 0.01 (-0.06 to 0.07)	0.841
a-tDCS + NCT	0.19 (0.13) vs. 0.15 (0.10)	0.04 (-0.04 to 0.11)	0.267
s-tDCS + NCT	0.13 (0.13) vs. 0.13 (0.14)	< 0.01 (-0.11 to 0.11)	0.981
NCT	0.20 (0.10) vs. 0.16 (0.07)	0.05 (-0.01 to 0.11)	0.218
Go/No-Go: ACCFA-Food§			
a-tDCS	0.10 (0.15) vs. 0.12 (0.13)	-0.02 (-0.08 to 0.03)	0.600
a-tDCS + NCT	0.13 (0.20) vs. 0.12 (0.07)	< 0.01 (-0.11 to 0.12)	0.951
s-tDCS + NCT	0.13 (0.21) vs. 0.05 (0.06)	0.08 (-0.06 to 0.23)	0.089
NCT	0.09 (0.07) vs. 0.07 (0.05)	0.02 (-0.02 to 0.06)	0.722
TMS: SICI§			
a-tDCS	0.50 (0.29) vs. 0.73 (0.41)	-0.23 (-0.50 to 0.03)	0.023
a-tDCS + NCT	0.57 (0.23) vs. 0.71 (0.25)	-0.14 (-0.34 to 0.07)	0.142
s-tDCS + NCT	0.56 (0.29) vs. 0.49 (0.42)	0.07 (-0.14 to 0.28)	0.479
NCT	0.55 (0.15) vs. 0.73 (0.26)	-0.18 (-0.33 to -0.03)	0.083

ACCFA = accuracy of false alarms; ANOVA = analysis of variance; a-tDCS = active transcranial direct current stimulation; FCQ = Food Craving Questionnaire; NCT = nutritional counseling therapy; SICI = short-interval intracortical inhibition; s-tDCS = sham transcranial direct current stimulation; TFEQ-21 = Three Factor Eating Questionnaire; TMS = transcranial magnetic stimulation.

† Bonferroni-corrected p-value for the within-group pre- to post-intervention effect from a mixed ANOVA.

‡ Non-parametric variables were compared using the Kruskal-Wallis rank order test.

§ Parametric variables were compared using ANOVA.
