# Erratum: Effect of home-based transcranial direct current stimulation combined with nutritional counseling therapy on binge eating disorder symptoms: a randomized pilot trial

**DOI:** 10.47626/1516-4446-2025-0508

**Published:** 2025-12-18

http://doi.org/10.47626/1516-4446-2025-0508

Jessica Lorenzzi Elkfury and Luciana C. Antunes, first authors of the article titled “Effect of home-based transcranial direct current stimulation combined with nutritional counseling therapy on binge eating disorder symptoms: a randomized pilot trial” (http://doi.org/10.47626/1516-4446-2024-3776), published in final form in volume 47 (2025), informed us of some errors in the article, which will be described below.

1) First, it should be noted that the two first authors, Jessica Lorenzzi Elkfury and Luciana C. Antunes, have equally contributed to this work and therefore share first authorship. Therefore, in the author byline, where it read:

Jessica Lorenzzi Elkfury,^1,2^https://orcid.org/0000-0002-5917-7199 Luciana C. Antunes,^2,3^https://orcid.org/0000-0002-0106-0721 Betina Franceschini Tocchetto,^1,2^ (...)

It should read:

Jessica Lorenzzi Elkfury,^1,2^*https://orcid.org/0000-0002-5917-7199 Luciana C. Antunes,^2,3^*https://orcid.org/0000-0002-0106-0721 Betina Franceschini Tocchetto,^1,2^ (...)

2) Consequently, at the end of the author institutional information, where it read:

(...) ^9^ Departamento de Cirurgia, Faculdade de Medicina, UFRGS, Porto Alegre, RS, Brazil.

It should read:

(...) ^9^ Departamento de Cirurgia, Faculdade de Medicina, UFRGS, Porto Alegre, RS, Brazil. * These authors have contributed equally to this manuscript.

3) Another error was found in the Methods section, Interventions subsection, subtitle “Home-based transcranial direct current stimulation.” Where it read:

The a-tDCS group received the current for 20 minutes (fade in/out: 10 seconds). The s-tDCS group received the same protocol, but the current stimulation automatically turned off 20 seconds after the stimulation had begun.

It should read:

The a-tDCS group received the current for 20 minutes. The s-tDCS group received only 30 seconds of stimulation at the beginning, midpoint, and end of the session.

Approved by Andre Brunoni, Editor-in-Chief.

